# Vestibular dysfunction in *NF2*–related schwannomatosis

**DOI:** 10.1093/braincomms/fcad089

**Published:** 2023-03-23

**Authors:** Amsal S Madhani, Susan King, Jennifer Zhu, Faisal Karmali, D Bradley Welling, Wenli Cai, Justin T Jordan, Richard F Lewis

**Affiliations:** Department of Otolargynology, Massachusetts Eye and Ear, Boston, MA, USA; Department of Otolargynology, Massachusetts Eye and Ear, Boston, MA, USA; Department of Otolargynology, Massachusetts Eye and Ear, Boston, MA, USA; Department of Otolargynology, Massachusetts Eye and Ear, Boston, MA, USA; Department of Otolaryngology Head and Neck Surgery, Harvard Medical School, Boston, MA, USA; Department of Otolargynology, Massachusetts Eye and Ear, Boston, MA, USA; Department of Otolaryngology Head and Neck Surgery, Harvard Medical School, Boston, MA, USA; Department of Neurology, Massachusetts General Hospital, Boston, MA, USA; Department of Radiology, Harvard Medical School, Boston, MA, USA; Department of Neurology, Massachusetts General Hospital, Boston, MA, USA; Department of Neurology, Harvard Medical School, Boston, MA, USA; Department of Otolargynology, Massachusetts Eye and Ear, Boston, MA, USA; Department of Otolaryngology Head and Neck Surgery, Harvard Medical School, Boston, MA, USA; Department of Neurology, Massachusetts General Hospital, Boston, MA, USA; Department of Neurology, Harvard Medical School, Boston, MA, USA

**Keywords:** vestibular, schwannoma, neurofibromatosis, balance, dizziness

## Abstract

*NF2*–related schwannomatosis is a genetic disorder characterized by neurologic tumours, most typically vestibular schwannomas that originate on the vestibulo-cochlear nerve(s). Although vestibular symptoms can be disabling, vestibular function has never been carefully analysed in *NF2*–related schwannomatosis. Furthermore, chemotherapy (e.g. bevacizumab) can reduce tumour volume and improve hearing in *NF2*–related schwannomatosis, but nothing is known about its vestibular effects. In this report, we studied the three primary vestibular-mediated behaviours (eye movements, motion perception and balance), clinical vestibular disability (dizziness and ataxia), and imaging and hearing in eight untreated patients with *NF2*–related schwannomatosis and compared their results with normal subjects and patients with sporadic, unilateral vestibular schwannoma tumours. We also examined how bevacizumab affected two patients with *NF2*–related schwannomatosis. Vestibular schwannomas in *NF2*–related schwannomatosis degraded vestibular precision (inverse of variability, reflecting a reduced central signal-to-noise ratio) but not vestibular accuracy (amplitude relative to ideal amplitude, reflecting the central signal magnitude) and caused clinical disability. Bevacizumab improved vestibular precision and clinical disability in both patients with *NF2*–related schwannomatosis but did not affect vestibular accuracy. These results demonstrate that vestibular schwannoma tumours in our *NF2*–related schwannomatosis population degrade the central vestibular signal-to-noise ratio, while bevacizumab improves the signal-to-noise ratio, changes that can be explained mechanistically by the addition (schwannoma) and suppression (bevacizumab) of afferent neural noise.

## Teaser

Madhani *et al*. assessed vestibular dysfunction in patients with neurofibromatosis type 2–related schwannomatosis. Vestibular schwannomas and bevacizumab, respectively, degraded and improved vestibular precision without affecting vestibular accuracy, consistent with a novel mechanism whereby afferent neural noise is generated by schwannoma(s) growing on the vestibular nerve and is suppressed by bevacizumab.

## Graphical Abstract

**Graphical abstract:**
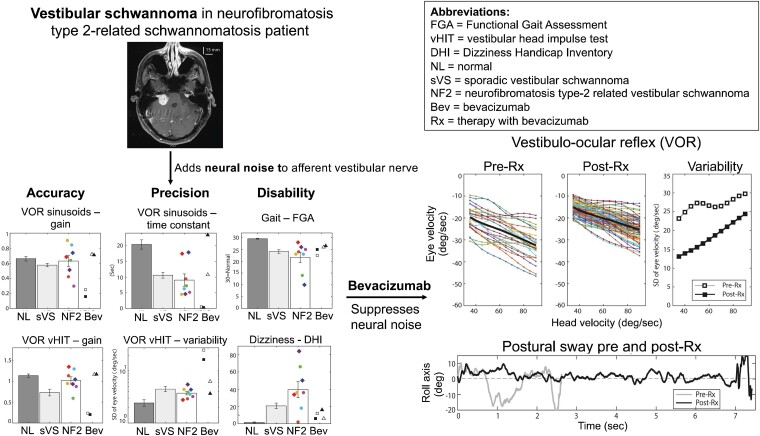

## Introduction

*NF2*–related schwannomatosis^1^ (NF2-SWN) is a genetic disease characterized by vestibular schwannomas (VSs) that originate from Schwann cells on the vestibular division of the vestibulo-cochlear nerve(s), and by neurologic tumours such as menigiomas and ependymomas.^2^ Previously called “neurofibromatosis type 2 (NF2)”, this disorder was recently renamed to emphasize the clinical and genetic overlap between neurofibromatosis and schwannomatosis.^1^ Although VS in patients with NF2-SWN (which we abbreviate as VS/NF2-SWN) damage the vestibular nerve^3^ and the vestibule and can cause disabling vestibular deficits,^4^ little research has been done on vestibular dysfunction in these patients.^5,6^ Furthermore, treatment with vascular endothelial growth factor (VEGF) inhibitors such as bevacizumab can reduce tumour volume and improve hearing in VS/NF2-SWN,^7^ but nothing is known about their effects on vestibular impairment. Here, we describe vestibular function in eight untreated patients with VS/NF2-SWN and in two patients with VS/NF2-SWN before and after bevacizumab. The *accuracy* and *precision* of the primary vestibular-mediated behaviours^8^ were quantified, where accuracy is the mean response amplitude relative to the ideal response, and precision is the inverse of trial-by-trail variability.^9^ Accuracy characterizes the *magnitude* of central vestibular signals, while precision characterizes the central *signal-to-noise ratio* (SNR), where signal is defined as neural activity that encodes vestibular information and noise is random neural activity.^10^ We also assessed clinical vestibular disability (dizziness and ataxia) and non-vestibular (MRI, audiologic) characteristics. Vestibular function in patients with VS/NF2-SWN was compared with that in normal subjects and patients with sporadic, unilateral VS tumours. We found that VS in NF2-SWN worsened vestibular precision (e.g. reduced the central SNR) and clinical disability, bevacizumab improved vestibular precision (e.g. increased the central SNR) and clinical disability, and neither VS nor bevacizumab affected vestibular accuracy.

## Materials and methods

The study was approved by the Massachusetts General Hospital and Massachusetts Eye and Ear Institutional Review Board and all patients signed informed consent prior to participation as per the Declaration of Helsinki. Ten patients with *VS/NF2-SWN* were recruited from the Massachusetts General Hospital Neurofibromatosis Clinic—eight had no prior intervention and were studied once; two patients were studied before and after a course of bevacizumab and had prior treatment (Table 1). Thirty-eight patients with *sporadic, unilateral VS* (*sVS*) were recruited from the Massachusetts Eye and Ear Otology clinic and were studied in an identical manner, as were 23 *normal* subjects. Exclusionary criteria were other otologic or neurologic disorders (except presbycusis or migraine); psychiatric disorders other than anxiety or depression; use of vestibular suppressants (benzodiazepines, antihistamines) and corrected visual acuity worse than 20/20.

**Table 1:** Clinical information for the *NF2*-related schwannomatosis patients

Patient ID	Age	Sex	VS side (U/B)	Other tumours	Genetic severity score	Years since dx	Prior VS therapies
Clinical information—untreated VS/NF2-SWN							
S1	26	F	U	L trigeminal SWN, cervical SWN, thoracic meningioma	1B, Mosaic NF2 c.193C > T	6	None
S2	63	F	B	None	Not performed	4	None
S3	17	F	B	Retroperitoneal SWN	1A	4	None
S4	39	F	U	R vagal SWN, cauda equina SWN	1A	6	None
S5	57	F	B	Right trigeminal SWN, bilateral vagal SWN, intracranial meningioma, thoracic spine ependymoma	1A	6	None
S6	49	F	B	None	2A, NF2:c.600–3C > A	4	None
S7	17	M	U	Subcostal SWNs, cutaneous SWN	1B, mosaic NF2:c.1021C > T	2	None
S8	59	M	B	None	Not performed	4	None
Clinical information—VS/NF2-SWN patients receiving bevacizumab							
S9	32	M	B (LVS resected)	Cervical meningioma	1B, Mosaic NF2:c.886-18_900del33	11	LVS resection 2010, Everolimus, bevacizumab
S10	29	F	B	Thoracic SWN	Not performed	6	Bevacizumab

B, bilateral; LVS resection, left vestibular schwannoma resection; SWN, schwannoma; U, unilateral.

### Vestibular evaluation

*Vestibular disability* was quantified using the dizziness handicap inventory (DHI) to measure dizziness severity [scaled from 0 (normal) to 100 (worst)];^11^ and the functional gait assessment (FGA) to measure gait dysfunction [scaled from 0 (worst) to 30 (normal)].^12^

The *vestibulo-ocular reflex* (*VOR*) was characterized using two methods we recently described:^13^ sinusoidal, yaw-axis, en-bloc rotations were performed over a 0.01–1.0 Hz range, and slow-phase eye movements were fitted to a sine function, yielding frequency-dependent gain, phase and bias values. These were fitted across frequencies to generate gain, time and bias constants.^13,14^ Passive head-on-body, impulsive (vestibular head impulse test, vHIT) yaw-axis rotations had a period of about 0.2 s, and VOR gain and variability were calculated separately for rotations towards the left and right. Gain was calculated as the mean slope of the eye versus head velocity plot, and VOR variability was the standard deviation across individual trials.^15^ VOR data from the control groups (sVS and normal subjects) were previously published,^13^ but VOR variability was recalculated for the current study since our prior study quantified VOR variability using a different method.

*Balance* was quantified as the longest time subjects could stand on foam with feet together and eyes closed without falling (e.g. taking a step) relative to the 24 s task duration,^16^ and trunk sway was measured with a 6 degree-of-freedom sensor affixed to the back. *Perceptual thresholds* for yaw-axis rotation were calculated with a direction-discrimination psychophysical task we previously described,^17^ using bell-shaped velocity profiles lasting 1 s.

### MRI evaluation

VS/NF2-SWN brain images were reviewed by one author (DBW) who tabulated VS location, volume, the presence or absence of brain compression, estimated mobility and FLAIR signal prominence in the cochlea and vestibule (see Table 2 for details).^18^ Apparent diffusional coefficient (ADC) values have been associated with VS responsiveness to VEGF inhibitor therapy^19^ in NF2-SWN patients, so these were calculated for the larger tumour (LT) by one author (WC) when adequate data were available. These values (Table 2) could be determined for four of the eight untreated patients and both of the patients who received bevacizumab (before and after therapy).

**Table 2:** Audiology and imaging information for the *NF2*-related schwannomatosis patients

Patient ID	PTA (ST)	PTA (LT)	WRS (ST)	WRS (LT)	VS Volume (ST)	VS Volume (LT)	VS Location (ST)	VS Location (LT)	CPA/IAC (ST)	CPA/IAC (LT)	Mobility (ST)	Mobility (LT)	FLAIR (ST)	FLAIR (LT)	ADC (LT)	
Audiology and imaging—untreated VS/NF2-SWN																
S1	2	5	98	92	0**−**	7567**+**	No tumour	CPA		40:1		2		C-1, V-0	1254	
S2	5	38	100	68	182**−**	14 850**+**	IAC	CPA, IAC	0	75:1	1	2	C-1, V-1	C-1, V-1	1143	
S3	3	40	98	74	156**−**	184**−**	IAC	IAC	0	0	0	0	C-2, V-1	C-2, V-1	n/a	
S4	10	25	96	96	0**−**	3863**+**	No tumour	CPA, IAC		27:1		2	C-1, V-1	C-1, V-1	1198	
S5	55	27	100	100	30**−**	86**−**	IAC	IAC	0	0	0	0	C-1, V-1	C-2, V-2	n/a	
S6	8	91	96	41.7	220**−**	9070**+**	IAC	CPA, IAC	0	870:1	0	2	n/a	n/a	1197	
S7	13	19	74	72	0**−**	390**−**	No tumour	IAC		0		0		C-0, V-0	n/a	
S8	76	55	36	72	466**−**	1397**−**	CPA	CPA, IAC	9:1	1:1	2	0	C-0, V-0	C-1, V-1	n/a	
**Patient ID**		**PTA (ST)**	**PTA (LT)**	**WRS (ST)**	**WRS (LT)**	**VS Volume (ST)**	**VS Volume (LT)**	**VS Location (ST)**	**VS Location (LT)**	**CPA/IAC (ST)**	**CPA/IAC (LT)**	**Mobility (ST)**	**Mobility (LT)**	**FLAIR (ST)**	**FLAIR (LT)**	**ADC (LT)**
Audiology & Imaging—VS/NF2-SWN patients receiving Bevacizumab																
S9	Pre-Rx	NFH	53	NFH	52	350**−**	5010**+**		CPA, IAC		12:1	0	0		C-0, V-0	1330
	Post-Rx	NFH	55	NFH	56	430**−**	4150**+**		CPA, IAC		9:1		n/a		n/a	1103
S10	Pre-Rx	55	27	8	100	1680**+**	7200**+**	CPA, IAC	CPA, IAC	8:1	2.5:1	0	0	C-2, V-2	C-2, V-1	1138
	Post-Rx	57	30	4	100	1540**+**	6180**+**	CPA, IAC	CPA, IAC	8:1	2.5:1	1	0	C-0, V-0	C-0, V-2	1224

ADC, apparent diffusional coefficient; CPA, cerebellopontine angle; CPA/IAC, ratio of tumour volume in each location; FLAIR, presence of abnormal MRI flair signal in the cochlea (C) or vestibular (V), graded from 0 (none) to 2 (most pronounced); IAC, internal auditory canal; LT, side with larger VS; Mobility, estimate of tumour mobility based on anatomic features, scored from 0 (least mobile) to 2 (most mobile); NFH, no functional hearing; PTA, pure tone average in dB; ST, side with smaller (or absent) VS. Note that S9 had a prior VS resection and vestibular neurectomy on the side labelled ST; VS Volume +, brain compression is present; VS Volume –, brain compression is absent; WRS, word recognition score in %.

### Hearing evaluation

Pure tone average (PTA) for air-conducted sound and word recognition scores (WRSs) for each ear were calculated using standard methods.

### Bevacizumab therapy

Patient S9 was tested 2 weeks after completing five 5 mg/kg infusions provided over 12 weeks; and Patient S10 was tested 3 months after completing five 15 mg/kg infusions provided over 12 weeks. Their treatment prior to this course of bevacizumab is summarized in Table 1.

### Statistical analysis

Data were normally distributed (Shapiro–Wilk test *P*-values > 0.05) except for the VOR time constant and variability, which were log-normally distributed. All comparisons therefore utilized standard parametric tests (ANOVA, *t*-tests, Pearson *R*) with VOR time constant and variability analysed in log units. Statistics were corrected for multiple comparisons using Bonferroni correction.

## Results

The eight untreated patients with VS/NF2-SWN (Table 1) had a mean age of 40.9 years (± 18.9 standard deviation), mean time since diagnosis of 4.5 years (± 1.4) and consisted of 6 females and 2 males. The 6 patients with genetic testing were classified in the 1A–2A range.^20^ Five patients had bilateral and three had unilateral VS. Non-VS tumours were present in five patients but these did not involve the ocular motor nerves and spinal tumours did not compress the cord. The control groups (normal, sVS) had mean ages of 44 (± 3.1 years) and 53 (± 1.8 years), and female:male ratios of 8:15 and 19:18, respectively. The VS/NF2-SWN population was comparable in age to the normal subjects but was younger than the sVS group (*P* = 0.02). The two patients with VS/NF2-SWN studied before and after bevacizumab were S9 (male, age 32 years) and S10 (female, age 29 years) and had undergone prior VS treatment (Table 1).

### Vestibular dysfunction in untreated VS/NF2-SWN

**Figure 1:**
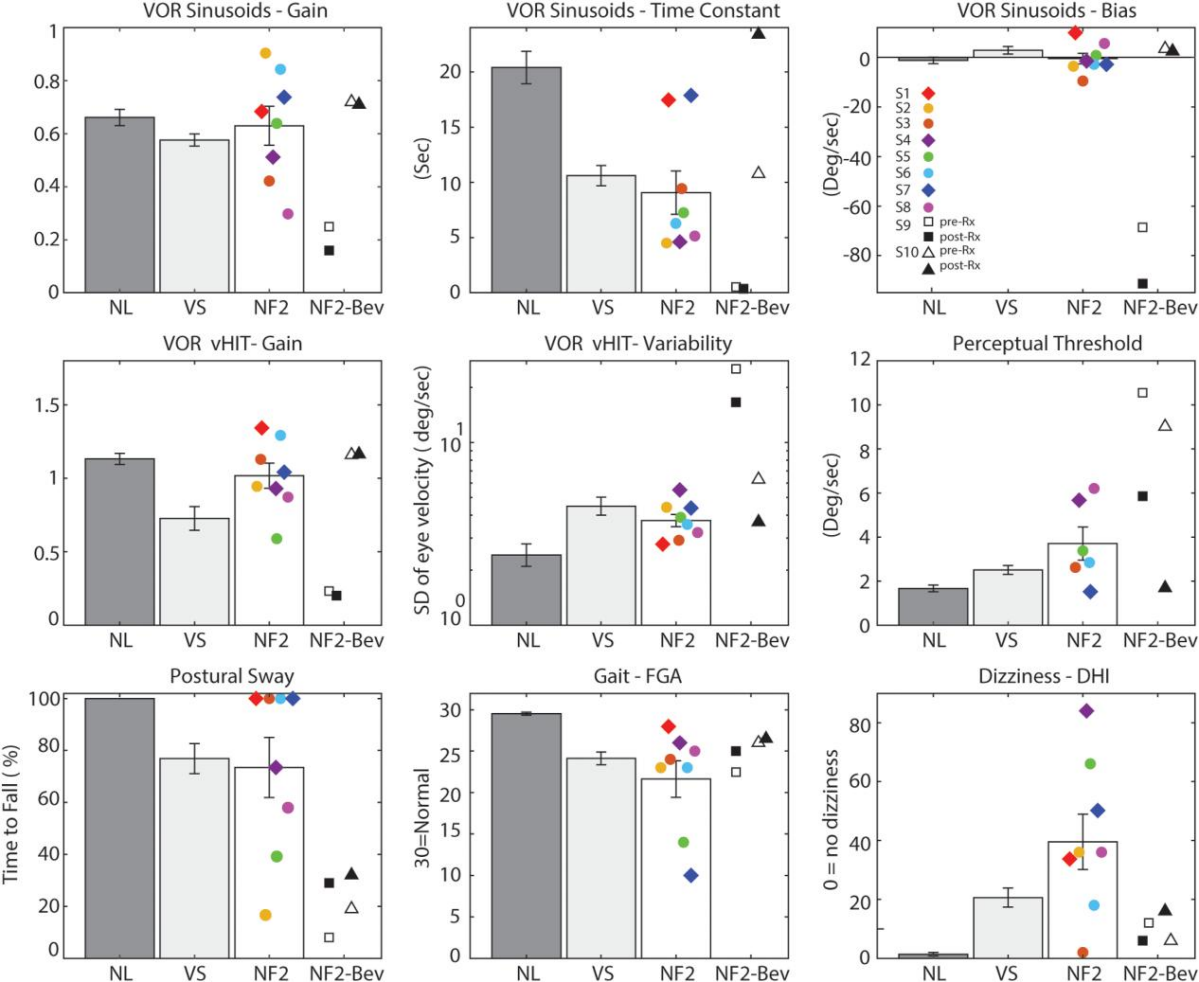
**Vestibular parameters in VS/NF2-SWN patients and control groups**. The mean ± 1 standard error are illustrated for the nine vestibular parameters, each of which is described in the Materials and methods. Dark grey bars denote normal subjects (NL, *n* = 23), light grey bars represent patients with sporadic vestibular schwannomas (VS, *n* = 38) and white bars denote the NF2-SWN group. Values for each of the eight untreated patients with NF2-SWN are illustrated with individual icons (see top right panel for subject legend). Patients with NF2-SWN with bilateral schwannomas are denoted by circles and patients with unilateral schwannomas are indicated by diamonds. Each panel also shows the results for the two patients with NF2-SWN tested before (open icons) and after (filled icons) the administration of bevacizumab, with S9 indicated by squares and S10 denoted by triangles.

Figure 1 shows the nine vestibular parameters in the VS/NF2-SWN, sVS and normal groups. These parameters can be grouped into four categories based on their pathophysiology,^9^ with: worse *precision* evidenced by higher VOR variability, greater postural sway (e.g. shorter time to fall), higher perceptual thresholds and shorter VOR time constants; worse *accuracy* evidenced by lower sinusoidal and vHIT VOR gains; worse *symmetry* evidenced by a larger VOR bias; and worse *clinical disability* evidenced by higher DHI and lower FGA scores.

### VS/NF2-SWN patients compared with control groups

Vestibular parameters in VS/NF2-SWN varied considerably between patients (Fig. 1), but taken as a group, *precision* metrics were worse in VS/NF2-SWN compared with normal subjects (ANOVA *P* = 0.003), with three of the four individual precision tests abnormal in VS/NF2-SWN (VOR time constant *P* < 0.008, perceptual threshold *P* = 0.01, sway *P* = 0.04). *Disability* metrics were also worse in patients with VS/NF2-SWN compared with normal subjects, both taken together (ANOVA *P* = 0.001) and individually (FGA *P* = 0.002, DHI *P* = 0.002). In contrast, *accuracy* measurements were normal in VS/NF2-SWN (sinusoidal VOR gain *P* = 0.65, vHIT gain *P* = 0.22).

*Precision* and *accuracy* metrics were equivalent in patients with VS/NF2-SWN and sVS (precision ANOVA *P* = 0.4; accuracy ANOVA *P* = 0.26), although precision metrics were qualitatively worse in VS/NF2-SWN for three of the four parameters and accuracy metrics were qualitatively better in VS/NF2-SWN for both parameters (Fig. 1). *Disability* was worse in VS/NF2-SWN than sVS (ANOVA *P* = 0.03), primarily because DHI scores were much higher in VS/NF2-SWN (*P* = 0.02). Vestibular *symmetry* was biased towards the tumour in sVS, was null in patients with VS/NF2-SWN and within VS/NF2-SWN was biased towards the tumour in the unilateral patients but towards the smaller tumour (ST) in bilateral patients.

### Relationship between vestibular metrics, imaging and hearing

Correlations between vestibular metrics and disability parameters were notable for the vHIT VOR variability–DHI relationship (*P* = 0.02) but otherwise were not significant, which likely reflects the small VS/NF2-SWN population. Table 2 summarizes the MRI characteristics for these patients, with the two sides designated as LT and ST (smaller or absent tumour). *FLAIR signal in the LT vestibule* was correlated with the VOR time constant (*P* = 0.04) and the VOR vHIT gain (0.01). The presence of *bilateral* (circles in Fig. 1) *rather than unilateral* (squares in Fig. 1) VS was associated with worse precision metrics for the VOR time constant, perceptual threshold and postural sway (but not for VOR variability), although these differences were not significant. VS *volume* in unilateral patients and the LT and ST tumour volumes in bilateral patients were not associated with the severity of vestibular dysfunction, nor was the tumour *location*, presence or absence of *brain compression*, *ADC* histogram value or net tumour burden (LT + ST volume). Hearing parameters, including PTA and WRSs for the LT and ST ears, were not correlated with any of the vestibular parameters.

### Effects of bevacizumab on vestibular function

**Figure 2:**
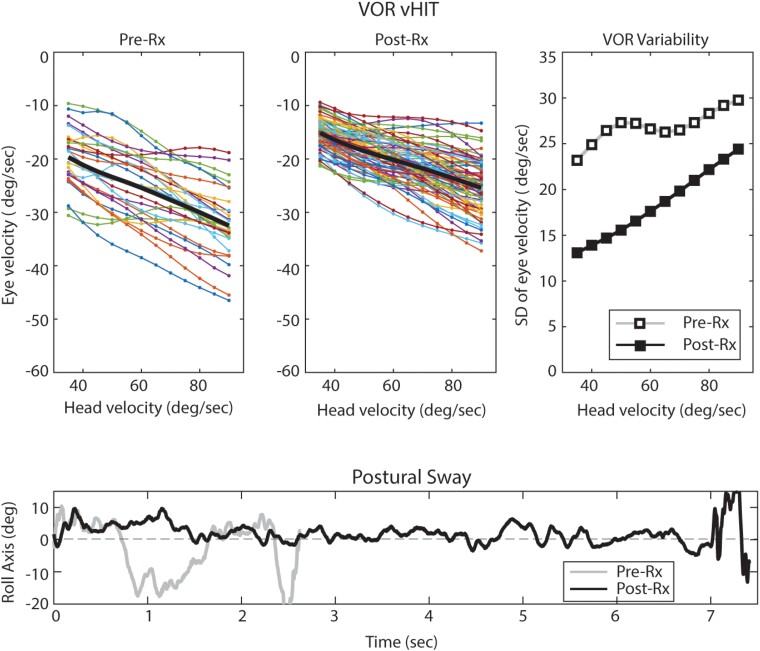
**Effect of bevacizumab on the VOR and postural stability**. The top row shows VOR responses produced by repeated impulsive head rotations in VS/NF2-SWN Patient S9, before (left panel) and after (middle panel) bevacizumab therapy. These plots show the eye velocity versus head velocity for each trial (thin lines) and the mean response (thick black line). VOR variability (the inverse of precision) is evidenced by the spread of eye velocities in these two panels and is quantified by the standard deviation (SD) across trials as a function of head velocity (right panel). The bottom row shows trunk sway about the earth-horizontal roll axis for VS/NF2-SWN Patient S10 before and after bevacizumab therapy, measured with a 6-degree of freedom (Shimmer) sensor affixed to the back. The stance condition was feet together, eyes closed, standing on compliant foam. Traces terminate when a ‘fall’ occurred, for example taking a step or opening the eyes.

**Figure 3:**
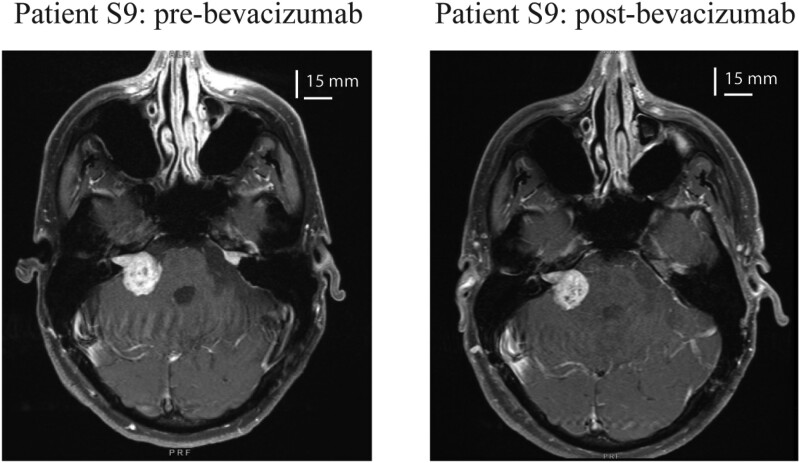
**Effect of bevacizumab on tumour size**. Brain MRIs on Patient S9 before and after receiving bevacizumab therapy. VS volume decreased by 17% after this treatment, which was associated with a small reduction in the compression of the brainstem and cerebellum by the tumour.

*Bevacizumab improved vestibular precision* in S9 and S10 (Figs 1 and 2) as evidenced by reduced VOR variability, reduced perceptual thresholds and improved balance for both patients, and an increased VOR time constant for S10. In contrast, *vestibular accuracy* (VOR gains) did not improve in either subject. Of note, S10 had normal VOR gains prior to bevacizumab but S9 (who had a prior unilateral vestibular neurectomy) had low gains and still did not improve. The FGA *disability measure* improved modestly in both patients and the DHI improved for S9 but not S10. *Symmetry* changes were inconsistent after treatment (worse in S9, no change in S10). *MRI imaging* (Table 2) showed that VS volumes decreased after treatment in the three tumour ears (e.g. the larger VS shrunk by 17% in S9 and 14% in S10), resulting in a modest reduction in the brainstem compression caused by the LT (Fig. 3). MRI FLAIR signal improved for both cochleas and for one of the two vestibules in S10; post-treatment FLAIR images were not available for S9 but pre-treatment images showed no abnormal FLAIR signal. ADC histogram value was higher in S9 than in S10 before bevacizumab and was reduced by treatment in S9, suggesting that tumour of the S9 could be considered to be more susceptible to chemotherapy than tumour of the S10.^19^ No difference was observed, however, between the two treated patients in the extent of VS shrinkage caused by bevacizumab or in the beneficial effects of the drug on vestibular function. *Hearing* data (Table 2) show that the PTA did not improve in the three hearing ears and the WRS improved slightly in one of the three hearing ears.

## Discussion

This brief report presents a detailed analysis of vestibular function in a small population of patients with VS/NF2-SWN and thereby provides the most *thorough description* of the vestibular consequences of VS in NF2-SWN. Our results also suggest that a *novel mechanism* is operative in VS/NF2-SWN, with VS tumours degrading function by generating noise on vestibular afferents and VEGF inhibition improving function by suppressing afferent noise.

### Pathophysiologic effects of VS and VEGF inhibition

Several mechanisms could potentially contribute to the pattern of vestibular dysfunction we observed. The *addition of afferent noise by the VS and suppression of afferent noise by bevacizumab* readily explains our findings—adding noise reduces the SNR (impairs precision), suppressing noise increases the SNR (improves precision), and neither affects signal magnitude (accuracy), exactly as we observed. Conversely, *reduction of afferent signal by the VS and recovery of signal by bevacizumab* is improbable since this putative mechanism requires that the brain normalize the central signal magnitude^21^ after the peripheral signal is reduced by the VS and increased by bevacizumab; and that signal changes in the vestibular periphery are not accompanied by similar changes in afferent noise. If the VS reduced signal and noise by roughly equivalent amounts, as would be expected with axonal death,^22^ for example, changes in the central SNR would be modest and could not explain our results.^23^

Mechanisms that depend on the *anatomic characteristics* of the VS tumours (Table 2) appear unlikely to explain the pattern of vestibular findings we observed, since no anatomic feature (tumour volume, location, ADC histogram analysis, estimated tumour motility or the presence or absence of brain compression) correlated with vestibular metrics in the untreated population. However, tumour location [e.g. internal auditory canal (IAC) versus cerebellopontine angle (CPA)] and volume may become more important mechanistically in patients with NF2-SWN who have more advanced disease and larger VS tumour.^24^ Gliosis of the vestibular nerve in the IAC^25^ and brain compression caused by LTs in the CPA, for example, could impair peripheral and central vestibular function, respectively, and thereby alter vestibular precision, accuracy and disability. In the two treated patients, contributions to improved vestibular function from tumour shrinkage and brain decompression cannot be excluded, but these structural changes were modest in size and it is improbable that they would affect vestibular precision (SNR) while sparing vestibular accuracy (signal magnitude).

Taken together, the above discussion suggests that the vestibular findings in our VS/NF2-SWN subjects were primarily caused by the *addition of afferent noise by the VS and the suppression of afferent noise by bevacizumab*. Mechanistically, changes in afferent noise mediated by the tumour and VEGF inhibitor could result in many possible effects. In particular, alterations in the function of the vestibular nerve and/or the labyrinth could be due to changes in vascular permeability,^26^ toxin release by the VS,^27^ changes in axonal myelination,^28^ and cellular inflammation mediated by the tumour’s micro-environment.^29^ Future work could examine the relationship between clinical vestibular dysfunction in patients with NF2-SWN and these putative mechanism by measuring tumour and serum biomarkers^30^ in addition to metrics that quantify vestibular function and disability.

Contrasting with our results, two prior studies of vestibular function in VS/NF2-SWN described reduced caloric and vestibular evoked myogenic potential (VEMP) amplitudes,^5,6^ evidence of reduced central vestibular signal magnitudes (vestibular precision was not measured in these studies). Notably, our sVS population had impaired vestibular precision similar to our patients with VS/NF2-SWN, but the patients with sVS also had abnormally low VOR gains,^13^ while our patients with VS/NF2-SWN did not. The patients with VS/NF2-SWN in our study were relatively young and their genetic severity scores were in the mild–moderate range (Table 1),^20^ while our sVS population consisted of older pre-surgical patients whose tumours were either growing or large enough to necessitate resection.^31^ Taken together, these observations imply that patients with VS/NF2-SWN with mild–moderate disease severity have impaired vestibular precision and normal vestibular accuracy, but older patients and/or those with larger or more aggressive tumours also have reduced vestibular accuracy. In our VS/NF2-SWN data, for example, the prominence of FLAIR signal in the vestibule^24^ was correlated with the VOR time constant (precision) and gain (accuracy), with the former likely reflecting hair cell damage and the latter hair cell death. The transition from impaired precision/normal accuracy to impaired precision/impaired accuracy, therefore, is probably due to the more extensive hair cell and axonal death in older patients^32^ or those with more severe disease.^33^

### Clinical correlations

The effects of VS tumours and VEGF inhibitors on vestibular precision generally paralleled their effects on clinical vestibular disability. We quantified disability using the FGA for gait and the DHI for dizziness, both of these metrics were impaired in untreated patients with VS/NF2-SWN, and three of four improved after bevacizumab for the two treated patients. Correlations between precision and disability metrics in the untreated patients with VS/NF2-SWN were present but were only significant for the vHIT VOR-DHI relationship, presumably because our patient population was small. In contrast, the larger sVS group we studied previously demonstrated prominent correlations between VOR precision and vestibular disability,^13^ so similar correlations are predicted when larger VS/NF2-SWN populations are studied. A relationship between precision and disability is not necessarily causal; however, since it could represent a co-dependence on underlying vestibular factors, we did not assay.

Future work should clarify the basis of clinical vestibular dysfunction in VS/NF2-SWN by testing a larger population of untreated and treated patients, expanding the metrics used to assess vestibular disability and adding tests that examine the integrity of all five vestibular end-organs and their innervation, rather than focusing on lateral canal (yaw rotation) function, which dominates our current approach. Both precision and accuracy can be gleaned, for example, from clinical tests of the vertical canals (using vHIT) and the otolith organs (using VEMP),^34^ and vestibular precision can also be quantified for the different vestibular end-organs and their sensory pathways using perceptual thresholds.^35^ In addition to vestibular, hearing and imaging measurements, assaying the concentration of secreted toxins^27^ could help determine their contribution(s) to the vestibular dysfunction in NF2-SWN caused by VS tumours and improved by VEGF inhibition.

## Conclusion

In our population of patients with VS/NF2-SWN, VS tumour(s) degraded vestibular precision and caused clinical disability, bevacizumab improved vestibular precision and alleviated clinical disability, but neither affected vestibular accuracy. These results indicate that the VS and bevacizumab, respectively, decreased and increased the central vestibular SNR without affecting the magnitude of the central vestibular signal. The most parsimonious explanation for these observations is a pathophysiologic mechanism in which aberrant neural noise on vestibular afferents is generated by the VS tumour(s) and suppressed by VEGF inhibition.

## Data Availability

Vestibular and auditory data are available at our laboratory’s website. Images are available through the Mass General Brigham radiology repository.

## Abbreviations

ADC =: apparent diffusional coefficient
CPA =: cerebellopontine angle
DHI =: dizziness handicap inventory
FGA =: functional gait assessment
IAC =: internal auditory canal
LT =: larger tumour
NF2-SWN =: *NF2*–related schwannomatosis
PTA =: pure tone average
SNR =: signal-to-noise ratio
ST =: smaller tumour
sVS =: sporadic vestibular schwannoma
VEGF =: vascular endothelial growth factor
VEMP =: vestibular evoked myogenic potential
vHIT =: vestibular head impulse test
VOR =: vestibulo-ocular reflex
VS =: vestibular schwannoma
WRS =: word recognition score
