# Correction to: Vestibular dysfunction in *NF2*–related schwannomatosis

**DOI:** 10.1093/braincomms/fcad179

**Published:** 2023-06-19

This is a correction to: Amsal S Madhani and others, Vestibular dysfunction in *NF2*–related schwannomatosis, *Brain Communications*, Volume 5, Issue 2, 2023, fcad089, https://doi.org/10.1093/braincomms/fcad089

The name of the disorder “Neurofibromatosis type 2-related schwannomatosis”, which was used throughout the manuscript upon original publication, has been corrected to “NF2-related schwannomatosis”.

